# Supplementary material for: Coordinated regulation of chemotaxis and resistance to copper by CsoR in Pseudomonas putida
Source: eLife. 2025 Apr 8;13:RP100914. doi: 10.7554/eLife.100914 (PMC11978298; doi:10.7554/eLife.100914)
Supplement: Supplementary file 1. — (a) Target proteins identified in the pull-down assay. (b) Strains and plasmids used in this work. (c) Primers used in this work. [file elife-100914-supp1.docx]

**Supplementary file 1a** Target proteins identified in a pull-down assay. iBAQ_T (%) and iBAQ_CK (%) represent the percentage of certain proteins in experimental and control samples, respectively. The *P* values are for three technical replicates. Protein names and descriptions are based on the annotated genome as indicated at http://www.pseudomonas.com.

| Protein name/ID | iBAQ_T (%) | iBAQ_CK (%) | Log_2_ (iBAQ_T  /iBAQ _CK) | *P* value | Description |
| --- | --- | --- | --- | --- | --- |
| CheA | 14.0253 | 0.0582 | 7.9126 | 0.0014 | Chemotaxis histidine kinase |
| CheY | 1.3015 | 0.0133 | 6.6104 | 0.0009 | Chemotaxis response regulator |
| CheZ | 0.5521 | 0.0280 | 4.3030 | 0.0039 | Protein phosphatase |
| PP_2359 | 0.0128 | 0.0004 | 5.0712 | 0.0071 | Putative Type 1 pili subunit CsuA/B protein |
| PP_3104 | 0.0506 | 0.0018 | 4.7829 | 0.0046 | Hypothetical protein |
| SspB | 0.3662 | 0.0182 | 4.3305 | 0.0191 | ClpXP protease specificity-enhancing factor |
| PP_1958 | 0.0135 | 0.0007 | 4.2978 | 0.0040 | Hypothetical protein |
| PP_1644 | 0.0227 | 0.0014 | 4.0341 | 0.0173 | NAD(P)H dehydrogenase |
| Eno | 0.0121 | 0.0007 | 4.0245 | 0.0390 | Enolase |
| GrpE | 0.0237 | 0.0017 | 3.7815 | 0.0156 | Heat shock protein GrpE |
| PaxB | 0.0314 | 0.0023 | 3.7735 | 0.0464 | Toxin secretion ATP-binding protein |
| ProB | 0.0092 | 0.0007 | 3.7289 | 0.0249 | Glutamate 5-kinase |
| PP_2907 | 0.0157 | 0.0013 | 3.6138 | 0.0209 | DNA-binding response regulator |
| CsoR | 0.0144 | 0.0012 | 3.6097 | 0.0149 | metal-binding protein |
| PP_1274 | 0.0367 | 0.0034 | 3.4318 | 0.0010 | Oxidoreductase, short-chain dehydrogenase |
| PP_0148 | 0.0126 | 0.0012 | 3.3665 | 0.0241 | Hypothetical protein |
| SpuD | 0.0026 | 0.0003 | 3.3282 | 0.0186 | Spermidine/putrescine ABC transporter substrate-binding protein |
| IspG | 0.0112 | 0.0012 | 3.2524 | 0.0051 | 4-hydroxy-3-methylbut-2-en-1-yl diphosphate synthase |
| GlnA | 0.0511 | 0.0057 | 3.1544 | 0.0360 | Glutamine synthetase |
| PP_3227 | 0.0041 | 0.0005 | 3.1500 | 0.0256 | Transcriptional regulator, LysR family |
| PP_5006 | 0.0139 | 0.0016 | 3.1473 | 0.0435 | Transcriptional regulator, TetR family |
| PP_3177 | 0.0055 | 0.0006 | 3.1171 | 0.0086 | Hypothetical protein |
| PP_3501 | 0.0017 | 0.0002 | 2.9666 | 0.0070 | Transposase |
| HflD | 0.0218 | 0.0029 | 2.9092 | 0.0142 | High frequency lysogenization protein HflD homolog |
| Pgl | 0.0246 | 0.0034 | 2.8652 | 0.0086 | 6-phosphogluconolactonase |
| MucA | 0.0186 | 0.0027 | 2.7673 | 0.0373 | Sigma factor AlgU negative regulatory protein |
| PP_3637 | 0.0101 | 0.0015 | 2.7629 | 0.0399 | Putative Sulfonate ABC transporter, ATP-binding protein |
| QuiP | 0.0022 | 0.0003 | 2.7169 | 0.0462 | Acyl-homoserine lactone acylase |
| PP_0568 | 0.0133 | 0.0022 | 2.6265 | 0.0054 | Y1_Tnp domain-containing protein |
| PP_4468 | 0.0133 | 0.0022 | 2.6077 | 0.0372 | Transcriptional regulator, Cro/CI family |
| PP_4460 | 0.0126 | 0.0023 | 2.4519 | 0.0406 | Transcriptional regulator, LysR family |
| PP_3640 | 0.0190 | 0.0036 | 2.4184 | 0.0236 | Transcriptional regulator, AraC family |
| PP_0120 | 0.0200 | 0.0038 | 2.4081 | 0.0181 | Putative zinc ABC transporter |
| NfuA | 0.0102 | 0.0019 | 2.4026 | 0.0262 | Fe/S biogenesis protein |
| PP_1489 | 0.0735 | 0.0147 | 2.3171 | 0.0248 | CheW domain protein |
| GlpR | 0.0371 | 0.0075 | 2.3065 | 0.0257 | DNA-binding transcriptional repressor |
| DctA | 0.0146 | 0.0031 | 2.2384 | 0.0269 | C4-dicarboxylate transport protein |
| MsrC | 0.0123 | 0.0027 | 2.1800 | 0.0488 | Free methionine-(R)-sulfoxide reductase |
| TatA-II | 0.0127 | 0.0028 | 2.1656 | 0.0168 | Sec-independent protein translocase protein |
| Gpx | 0.0123 | 0.0030 | 2.0279 | 0.0025 | Glutathione peroxidase |
| PP_4932 | 0.0293 | 0.0067 | 2.1332 | 0.0414 | Putative D-arabinose 1-dehydrogenase |
| FusB | 0.0052 | 0.0011 | 2.2250 | 0.0322 | Elongation factor G 2 |
| PP_2683 | 0.0029 | 0.0006 | 2.2718 | 0.0178 | Putative sensory box histidine kinase/response regulator |

**Supplementary file 1b** Strains and plasmids used in this work.

| Strain or plasmid | Relevant genotype and/or description | Source or reference |
| --- | --- | --- |
| *E. coli* strains |  |  |
| DH5a | λ-Φ80dlacZΔM15Δ(*lacZYA*-*argF*)*U196 recA1 endA1 hsdR17*(rK- mK -) *supE44 thi-1 gyrA relA1* | Invitrogen Corp |
| S17-1/λpir | *RP4-2*(Km::Tn7,Tc::Mu-1), *pro-82*, *LAMpir*, *recA1*, *endA1*, *thiE1*, *hsdR17*, *creC510*, host for *pir*-dependent plasmids | Invitrogen Corp |
| BL21(DE3) | F-, *ompT*, *hsdS*(*rBB*-*mB*－), *gal*, *dcm*(DE3) | Invitrogen Corp |
| BTH101 | F-, cya-*99*, *araD139*, *galE15*, *galK16*, *rpsL1*, *hsdR2*, *mcrA1*, *mcrB1*, host for bacterial two-hybrid assay (BTH) | Stratagene |
| *P. putida* strains |  |  |
| WT | Wild type KT2440 | Lab stock |
| WT+pVec | Wild type KT2440 harboring empty vector pBBR1MCS-5 | Lab stock |
| WT+p*cheA* | Wild type KT2440 harboring empty vector pBBR1MCS-5-*cheA* | Lab stock |
| WT+p*csoR* | Wild type KT2440 harboring empty vector pBBR1MCS-5-*csoR* | Lab stock |
| Δ*csoR* | Unmarked *csoR* (*PP_2969*) deletion mutant | This work |
| Δ*csoR*+pVec | Δ*csoR* strain harboring empty vector pBBR1MCS-5 | This work |
| Δ*csoR*+p*csoR* | Δ*csoR* strain complemented with pBBR1MCS-5-*csoR* | This work |
| Δ*cheA* | Unmarked *cheA* (*PP_4338*) deletion mutant | This work |
| Δ*cheA*+pVec | Δ*cheA* strain harboring empty vector pBBR1MCS-5 | This work |
| Δ*cheA*+p*cheA* | Δ*cheA* strain complemented with pBBR1MCS-5-*cheA* | This work |
| Δ*cheA*Δ*csoR* | Unmarked *cheA csoR* double deletion mutant | This work |
| Δ*cheA*Δ*csoR*+pVec | Δ*cheA*Δ*csoR* strain harboring empty vector pBBR1MCS-5 | This work |
| Δ*cheA*Δ*csoR*+p*cheA* | Δ*cheA*Δ*csoR* strain complemented with pBBR1MCS-5-*cheA* | This work |
| Δ*cheA*Δ*csoR*+p*csoR* | Δ*cheA*Δ*csoR* strain complemented with pBBR1MCS-5-*csoR* | This work |
| Δ*cheA*Δ*csoR*+p*cheA*-*csoR* | Δ*cheA*Δ*csoR* strain complemented with pBBR1MCS-5-*cheA-csoR* | This work |
| Plasmids |  |  |
| pBBR401 | Knockout vector, derived from pBBR1-MCS5, replication fragment replaced by ori R6K replication fragment | Lab stock |
| pBBR401-*cheA*UP-DW | pBBR401 containing up and down homologous region of *cheA* | This work |
| pBBR401-*csoR*UP-DW | pBBR401 containing up and down homologous region of *csoR* | This work |
| pBBR1MCS5 | Expression vector containing gene coding α-subunit of *β*-galactosidase, Gm^r^ | Lab stock |
| pBBR1MCS5-*cheA* | pBBR1MCS5 containing intact *cheA* gene | This work |
| pBBR1MCS5-*cheA*_ΔHPT_ | pBBR1MCS5 containing truncated *cheA* without the region encoding HPT domain | This work |
| pBBR1MCS5-*cheA*_ΔYB_ | pBBR1MCS5 containing truncated *cheA* without the region encoding CheY binding domain | This work |
| pBBR1MCS5-*cheA*_ΔDim_ | pBBR1MCS5 containing truncated *cheA* without the region encoding dimerization domain | This work |
| pBBR1MCS5-*cheA*_ΔHATPase_ | pBBR1MCS5 containing truncated *cheA* without the region encoding HATPase domain | This work |
| pBBR1MCS5-*cheA*_ΔWB_ | pBBR1MCS5 containing truncated *cheA* without the region encoding CheW binding domain | This work |
| pBBR1MCS5-*csoR* | pBBR1MCS5 containing intact *csoR* gene | This work |
| pBBR1MCS5-*PP_5006* | pBBR1MCS5 containing intact *PP_5006* gene | This work |
| pET-28a | T7/his-tag expression vector, Km^r^ | Addgene |
| pHS-Strep | Protein expression vector with Strep II-tag, Km^r^ | This work |
| pKT-25a | Plasmid for BTH, Km^r^ | Lab stock |
| pKT-25a-*zip* | pKT-25a harboring *zip* gene, positive control in BTH | Lab stock |
| pUT-18C | Plasmid for bacterial two-hybrid, Amp^r^ | Lab stock |
| pUT-18C-*zip* | pUT-18C harboring *zip* gene, positive control in BTH | Lab stock |
| pBBR403-KN151-LC151 | Plasmid for bimolecular fluorescence complementation (BiFC), Gm^r^ | This work |
| pBBR403-Jun-KN151-Fos-LC151 | pBBR403-KN151-LC151 harboring Jun and Fos genes, positive control in BiFC | This work |

**Supplementary file 1c** Primers used in this work.

| Primers | Sequence^a^ |
| --- | --- |
| 28a-CheA s | TGGGTCGCGGATCCATGAGCTTCGGCGCCG |
| 28a-CheA a | TGGTGGTGCTCGAGCCACCACCAGGACCTTGA |
| T18/T25-*cheA* s | CTAGTGGTGAATTCATGAGCTTCGGCGCCG |
| T18/T25-*cheA* a | ATCTAGATCTCGAGCCACCACCAGGACCTTGA |
| T18/T25-*csoR* s | CTAGTGGTGAATTCATGAGCGATCACGAACACG |
| T18/T25-*csoR* a | ATCTAGATCTCGAGCAGAACAGCCTGCTTCACC |
| T18/T25-*PP_1188* s | CTAGTGGTGAATTCATGACGACACGTCAGCCG |
| T18/T25-*PP_1188* a | ATCTAGATCTCGAGGGTGAAGTTCTGGGATGTGC |
| T18/T25-*PP_1612* s | CTAGTGGTGAATTCATGGCAAAAATCGTCGAC |
| T18/T25-*PP_1612* a | ATCTAGATCTCGAGGCAAGAGCAGAACGAGGA |
| T18/T25-*PP_4111* s | CTAGTGGTGAATTCACAACGCCCATCGAGCTG |
| T18/T25-*PP_4111* a | ATCTAGATCTCGAGGCGGTGCAAGAAGCTGTTG |
| T18/T25-*PP_5046* s | CTAGTGGTGAATTCATGTCGAAGTCGGTTCAAC |
| T18/T25-*PP_5046* a | ATCTAGATCTCGAGATAGAGCATAGCGTGACAGG |
| T18/T25-*PP_1074* s | CTAGTGGTGAATTCGGACCGCCCATGAATCTG |
| T18/T25-*PP_1074* a | ATCTAGATCTCGAGGATTACGGCAAGGTCATAGCAG |
| T18/T25-*PP_1874* s | CTAGTGGTGAATTCATGGCTGCAACGATGCTG |
| T18/T25-*PP_1874* a | ATCTAGATCTCGAGCGACCTGGTGCTGATGGACT |
| T18/T25-*PP_4728* s | CTAGTGGTGAATTCGATGAGCAGCTGAACGAGA |
| T18/T25-*PP_4728* a | ATCTAGATCTCGAGCAGAATGGAGACGCACGA |
| T18/T25-*PP_4015* s | CTAGTGGTGAATTCCGCATGAGCAACCTGCAG |
| T18/T25-*PP_4015* a | ATCTAGATCTCGAGACCGCAGTGAGCGAAGAAA |
| T18/T25-*PP_0853* s | CTAGTGGTGAATTCATGCACGGCGAATCTCC |
| T18/T25-*PP_0853* a | ATCTAGATCTCGAGCGACTGTTCTGGCAGGATG |
| T18/T25-*PP_1877* s | CTAGTGGTGAATTCATGATCGACCTCAATGCCA |
| T18/T25-*PP_1877* a | ATCTAGATCTCGAGGCCGAAGGTGTATGAAGAGC |
| T18/T25-*PP_1428* s | CTAGTGGTGAATTCATGAGTCGTGAAGCTTTGCA |
| T18/T25-*PP_1428* a | ATCTAGATCTCGAGCTGCCGTTGCGTTCGTAG |
| T18/T25-*PP_2378* s | CTAGTGGTGAATTCATGAGCGCTATAACCATTACC |
| T18/T25-*PP_2378* a | ATCTAGATCTCGAGGAAACCATCCTTTACAGGGA |
| T18/T25-*PP_0167* s | CTAGTGGTGAATTCGTGGAATCCGAAGTCAGTC |
| T18/T25-*PP_0167* a | ATCTAGATCTCGAGCCCATACAATCAAGAACACG |
| T18/T25-*PP_1023* s | CTAGTGGTGAATTCATGGGAGGGCGTGGTATG |
| T18/T25-*PP_1023* a | ATCTAGATCTCGAGCCTTGAGGCCATGCTGTGA |
| T18/T25-*PP_0691* s | CTAGTGGTGAATTCATGCGAAGCAAGGTGACG |
| T18/T25-*PP_0691* a | ATCTAGATCTCGAGGAACTTGAACACGGTGGACA |
| T18/T25-*PP_1108* s | CTAGTGGTGAATTCTTTCCTCCCTTCCGCCT |
| T18/T25-*PP_1108* a | ATCTAGATCTCGAGTGGACGTTAGTTCGGATGG |
| T18/T25-*PP_5181* s | CTAGTGGTGAATTCATGAATAAAATGGGCAAGAC |
| T18/T25-*PP_5181* a | ATCTAGATCTCGAGTGCCGCAATCATCAATTA |
| T18/T25-*PP_1321* s | CTAGTGGTGAATTCAGCTTTAAGGAGCCGTTG |
| T18/T25-*PP_1321* a | ATCTAGATCTCGAGCGTCAAGAAGCCAACTCG |
| T18/T25-*PP_5016* s | CTAGTGGTGAATTCATGGGTATCTTTGACTGGAA |
| T18/T25-*PP_5016* a | ATCTAGATCTCGAGGCTTCCTCTTCCATCTGC |
| T18/T25-*PP_0120* s | CTAGTGGTGAATTCGTGTCCCGATTCCTGGCT |
| T18/T25-*PP_0120* a | ATCTAGATCTCGAGCGACTGGTGCCTGGATGA |
| T18/T25-*PP_0148* s | CTAGTGGTGAATTCGTGATGCAGGATGTAACCG |
| T18/T25-*PP_0148* a | ATCTAGATCTCGAGCCAACGTGAAGCAGATCG |
| T18/T25-*PP_0568* s | CTAGTGGTGAATTCATGCAACGTCCAGGCTCC |
| T18/T25-*PP_0568* a | ATCTAGATCTCGAGTGGCGTTTCTTGGGTTCAT |
| T18/T25-*PP_1274* s | CTAGTGGTGAATTCATGCCCACCGTCCTGATC |
| T18/T25-*PP_1274* a | ATCTAGATCTCGAGACAGCCTGTACTGGCCTCTT |
| T18/T25-*PP_1489* s | CTAGTGGTGAATTCGCGATGAACGACTTGCAA |
| T18/T25-*PP_1489* a | ATCTAGATCTCGAGGCAGCCAGTAATCGTCCAG |
| T18/T25-*PP_1644* s | CTAGTGGTGAATTCGGAGATCCTGCCGTGAGC |
| T18/T25-*PP_1644* a | ATCTAGATCTCGAGCAGGTTGTTCACCACCAGCA |
| T18/T25-*PP_1958* s | CTAGTGGTGAATTCCCAGCGAGGCAGAGGGT |
| T18/T25-*PP_1958* a | ATCTAGATCTCGAGCTTGCCTGCTCCTCCTGAT |
| T18/T25-*PP_2359* s | CTAGTGGTGAATTCATGCGAACGAACCTTTCA |
| T18/T25-*PP_2359* a | ATCTAGATCTCGAGAACACGCTGACGCCAGT |
| T18/T25-*PP_2683* s | CTAGTGGTGAATTCATGGCGAGGCCCTCTGA |
| T18/T25-*PP_2683* a | ATCTAGATCTCGAGTGGCGTTACTCCATTGTCAGA |
| T18/T25-*PP_2907* s | CTAGTGGTGAATTCATGCCTCGCGTACTGACC |
| T18/T25-*PP_2907* a | ATCTAGATCTCGAGTGGAGACCTCGAAGTACAGCAC |
| T18/T25-*PP_3104* s | CTAGTGGTGAATTCGGGCAAGACGGCCTTCTC |
| T18/T25-*PP_3104* a | ATCTAGATCTCGAGGCCAATATCACGACCAGCAG |
| T18/T25-*PP_3177* s | CTAGTGGTGAATTCCCTGTCTTTGTGCTTTTGC |
| T18/T25-*PP_3177* a | ATCTAGATCTCGAGGAGGCTGTCCTTGAACGAGT |
| T18/T25-*PP_3227* s | CTAGTGGTGAATTCGATGATATGGACGCCTTCG |
| T18/T25-*PP_3227* a | ATCTAGATCTCGAGGCGTTGCCTAAGACCGTATT |
| T18/T25-*PP_3501* s | CTAGTGGTGAATTCATGACTTTCTCGCCCAATC |
| T18/T25-*PP_3501* a | ATCTAGATCTCGAGCAGTCAGAAGCGAGTTATGG |
| T18/T25-*PP_3637* s | CTAGTGGTGAATTCGTGTTGCTGGAGGCCCG |
| T18/T25-*PP_3637* a | ATCTAGATCTCGAGGCCGTTGTTATGTGCCACC |
| T18/T25-*PP_3640* s | CTAGTGGTGAATTCATGATTTCGTCCAGCCCG |
| T18/T25-*PP_3640* a | ATCTAGATCTCGAGGTGGCTATTCGGCTTGCTG |
| T18/T25-*PP_4460* s | CTAGTGGTGAATTCATGGAGAACAGAATCACCCT |
| T18/T25-*PP_4460* a | ATCTAGATCTCGAGGCCTTCCACTCGTATCAGC |
| T18/T25-*PP_4468* s | CTAGTGGTGAATTCATGCAGCTGAGAATTGCC |
| T18/T25-*PP_4468* a | ATCTAGATCTCGAGGCCGAACTCGCTTACCA |
| T18/T25-*PP_4932* s | CTAGTGGTGAATTCATGAGCCTGCCAACCCTG |
| T18/T25-*PP_4932* a | ATCTAGATCTCGAGTTGCGGATCAGCGTTCG |
| T18/T25-*PP_5006* s | CTAGTGGTGAATTCGACTGGATGAAAACCCGC |
| T18/T25-*PP_5006* a | ATCTAGATCTCGAGGCTGCAACACTACATCTCCAG |
| BIFC CheA s | AGGAAACAGAATTCATGCGTTTGATGAGCTTCGGC |
| BIFC CheA a | CTCGAGCCGAGCTCGGCGTAACGCTTGAGCAT |
| BIFC CsoR s | AAACTAGTGGATCCATGAGCGATCACGAACAC |
| BIFC CsoR a | CCGATCGCTCTAGAGAGGTACTTAGTGATTTGCTTG |
| BIFC PP_1612 s | AAACTAGTGGATCCATGGCAAAAATCGTCGAC |
| BIFC PP_1612 a | CCGATCGCTCTAGAGCGAAACTCGGCACGACC |
| BIFC PP_4111 s | AAACTAGTGGATCCCGCACAACGCCCATCGAG |
| BIFC PP_4111 a | CCGATCGCTCTAGACCCGCGGCTCTTCTTGAC |
| BIFC PP_5046 s | AAACTAGTGGATCCATGTCGAAGTCGGTTCAA |
| BIFC PP_5046 a | CCGATCGCTCTAGAGCAGCTGTAGTACAGCTC |
| BIFC PP_1074 s | AAACTAGTGGATCCATGAATCTGCCCCCCCGC |
| BIFC PP_1074 a | CCGATCGCTCTAGAGACCACCTCAAGCCTGAT |
| BIFC PP_4728 s | AAACTAGTGGATCCATGGCTGATGAGCAGCTG |
| BIFC PP_4728 a | CCGATCGCTCTAGAAGCCTTTTCATTGATCGA |
| BIFC PP_0853 s | AAACTAGTGGATCCATGCACGGCGAATCTCCG |
| BIFC PP_0853 a | CCGATCGCTCTAGAGCCACGAGCGATCAACGC |
| BIFC PP_1877 s | AAACTAGTGGATCCATGATCGACCTCAATGCC |
| BIFC PP_1877 a | CCGATCGCTCTAGAGCAGTCAGTCAACGCCAG |
| BIFC PP_2378 s | AAACTAGTGGATCCATGAGCGCTATAACCATT |
| BIFC PP_2378 a | CCGATCGCTCTAGAGTAGGCGTTTTCTTTCTG |
| BIFC PP_1023 s | AAACTAGTGGATCCATGGGAGGGCGTGGTATG |
| BIFC PP_1023 a | CCGATCGCTCTAGATGGGCACCAGTAGATGTC |
| BIFC PP_0691 s | AAACTAGTGGATCCATGCGAAGCAAGGTGACG |
| BIFC PP_0691 a | CCGATCGCTCTAGATACCAGCACCAGGTTGTC |
| BIFC PP_0148 s | AAACTAGTGGATCCGTGATGCAGGATGTAACCG |
| BIFC PP_0148 a | CCGATCGCTCTAGACAGGCGCTCCTCATCTTC |
| BIFC PP_1644 s | AAACTAGTGGATCCGCGCCCTACATCCTGGTG |
| BIFC PP_1644 a | CCGATCGCTCTAGACGCCGCCTCCAGGGCCTT |
| BIFC PP_2683 s | AAACTAGTGGATCCATGGCGAGGCCCTCTGAC |
| BIFC PP_2683 a | CCGATCGCTCTAGAATGCACCAGGAGCAACTG |
| BIFC PP_3177 s | AAACTAGTGGATCCGTGCTTTTGCACAGCGCT |
| BIFC PP_3177 a | CCGATCGCTCTAGAAGTTGGCAACAGCTGCAA |
| BIFC PP_3227 s | AAACTAGTGGATCCGATGATATGGACGCCTTC |
| BIFC PP_3227 a | CCGATCGCTCTAGAACCCAACAACCGATGTCC |
| BIFC PP_3501 s | AAACTAGTGGATCCATGACTTTCTCGCCCAAT |
| BIFC PP_3501 a | CCGATCGCTCTAGAGGCAGGCACCCACTGATG |
| BIFC PP_4460 s | AAACTAGTGGATCCATGGAGAACAGAATCACC |
| BIFC PP_4460 a | CCGATCGCTCTAGAGCTTCGGATTAGCAGATG |
| BIFC PP_5006 s | AAACTAGTGGATCCGACTGGATGAAAACCCGC |
| BIFC PP_5006 a | CCGATCGCTCTAGACCCCTCCAGGTACTTCAC |
| 403-CsoR s | AGGAAACAGAATTCATGAGCGATCACGAACACG |
| 403-CsoR a | ACTCTAGAGGATCCCAGAACAGCCTGCTTCACC |
| 403-PP_1612 s | AGGAAACAGAATTCATGGCAAAAATCGTCGAC |
| 403-PP_1612 a | ACTCTAGAGGATCCGCAAGAGCAGAACGAGGA |
| 403-PP_4111 s | AGGAAACAGAATTCGTCCACCCACAGGTAGTCC |
| 403-PP_4111 a | ACTCTAGAGGATCCGGAGCGGTGCAAGAAGC |
| 403-PP_5046 s | AGGAAACAGAATTCATGTCGAAGTCGGTTCAAC |
| 403-PP_5046 a | ACTCTAGAGGATCCATAGAGCATAGCGTGACAGG |
| 403-PP_1074 s | AGGAAACAGAATTCGGACCGCCCATGAATCTG |
| 403-PP_1074 a | ACTCTAGAGGATCCGATTACGGCAAGGTCATAGCAG |
| 403-PP_4728 s | AGGAAACAGAATTCCTCGCAGTCCACAAAA |
| 403-PP_4728 a | ACTCTAGAGGATCCCCCGAAACTTGAATTTG |
| 403-PP_0853 s | AGGAAACAGAATTCATGCACGGCGAATCTCC |
| 403-PP_0853 a | ACTCTAGAGGATCCCGACTGTTCTGGCAGGATG |
| 403-PP_1877 s | AGGAAACAGAATTCATGATCGACCTCAATGCCA |
| 403-PP_1877 a | ACTCTAGAGGATCCGCCGAAGGTGTATGAAGAGC |
| 403-PP_2378 s | AGGAAACAGAATTCATGAGCGCTATAACCATTACC |
| 403-PP_2378 a | ACTCTAGAGGATCCGAAACCATCCTTTACAGGGA |
| 403-PP_1023 s | AGGAAACAGAATTCATGGGAGGGCGTGGTATG |
| 403-PP_1023 a | ACTCTAGAGGATCCCCTTGAGGCCATGCTGTGA |
| 403-PP_0691 s | AGGAAACAGAATTCATGCGAAGCAAGGTGACG |
| 403-PP_0691 a | ACTCTAGAGGATCCGAACTTGAACACGGTGGACA |
| 403-PP_0148 s | AGGAAACAGAATTCGTGATGCAGGATGTAACCG |
| 403-PP_0148 a | ACTCTAGAGGATCCCCAACGTGAAGCAGATCG |
| 403-PP_1644 s | AGGAAACAGAATTCCCGCCGGAAAAGGTGC |
| 403-PP_1644 a | ACTCTAGAGGATCCGCGATCAGGCCGAAGA |
| 403-PP_3177 s | AGGAAACAGAATTCCCTGTCTTTGTGCTTTTGC |
| 403-PP_3177 a | ACTCTAGAGGATCCGAGGCTGTCCTTGAACGAGT |
| 403-PP_3501 s | AGGAAACAGAATTCATGACTTTCTCGCCCAATC |
| 403-PP_3501 a | ACTCTAGAGGATCCCAGTCAGAAGCGAGTTATGG |
| 403-PP_5006 s | AGGAAACAGAATTCACGTGCTGACCCGATGA |
| 403-PP_5006 a | ACTCTAGAGGATCCCCTACAAGATGCGCCAAA |
| 28a-CsoR s | TGGGTCGCGGATCCATGAGCGATCACGAACACG |
| 28a-CsoR a | TGGTGGTGCTCGAGCAGAACAGCCTGCTTCACC |
| 28a-PP_1612 s | TGGGTCGCGGATCCATGGCAAAAATCGTCGAC |
| 28a-PP_1612 a | TGGTGGTGCTCGAGGCAAGAGCAGAACGAGGA |
| 28a-PP_4111 s | TGGGTCGCGGATCCACAACGCCCATCGAGCTG |
| 28a-PP_4111 a | TGGTGGTGCTCGAGGCGGTGCAAGAAGCTGTTG |
| 28a-PP_5046 s | TGGGTCGCGGATCCATGTCGAAGTCGGTTCAAC |
| 28a-PP_5046 a | TGGTGGTGCTCGAGATAGAGCATAGCGTGACAGG |
| 28a-PP_1074 s | TGGGTCGCGGATCCGGACCGCCCATGAATCTG |
| 28a-PP_1074 a | TGGTGGTGCTCGAGGATTACGGCAAGGTCATAGCAG |
| 28a-PP_4728 s | TGGGTCGCGGATCCGATGAGCAGCTGAACGAGA |
| 28a-PP_4728 a | TGGTGGTGCTCGAGCAGAATGGAGACGCACGA |
| 28a-PP_0853 s | TGGGTCGCGGATCCATGCACGGCGAATCTCC |
| 28a-PP_0853 a | TGGTGGTGCTCGAGCGACTGTTCTGGCAGGATG |
| 28a-PP_1877 s | TGGGTCGCGGATCCATGATCGACCTCAATGCCA |
| 28a-PP_1877 a | TGGTGGTGCTCGAGGCCGAAGGTGTATGAAGAGC |
| 28a-PP_2378 s | TGGGTCGCGGATCCATGAGCGCTATAACCATTACC |
| 28a-PP_2378 a | TGGTGGTGCTCGAGGAAACCATCCTTTACAGGGA |
| 28a-PP_1023 s | TGGGTCGCGGATCCATGGGAGGGCGTGGTATG |
| 28a-PP_1023 a | TGGTGGTGCTCGAGCCTTGAGGCCATGCTGTGA |
| 28a-PP_0691 s | TGGGTCGCGGATCCATGCGAAGCAAGGTGACG |
| 28a-PP_0691 a | TGGTGGTGCTCGAGGAACTTGAACACGGTGGACA |
| 28a-PP_0148 s | TGGGTCGCGGATCCGTGATGCAGGATGTAACCG |
| 28a-PP_0148 a | TGGTGGTGCTCGAGCCAACGTGAAGCAGATCG |
| 28a-PP_1644 s | TGGGTCGCGGATCCGATCCTGCCGTGAGCGCG |
| 28a-PP_1644 a | TGGTGGTGCTCGAGCAGGTTGTTCACCACCAGCA |
| 28a-PP_2683 s | TGGGTCGCGGATCCTTCATGGCGAGGCCCTCT |
| 28a-PP_2683 a | TGGTGGTGCTCGAGTGGCGTTACTCCATTGTCAGA |
| 28a-PP_3177 s | TGGGTCGCGGATCCCCTGTCTTTGTGCTTTTGC |
| 28a-PP_3177 a | TGGTGGTGCTCGAGGAGGCTGTCCTTGAACGAGT |
| 28a-PP_3227 s | TGGGTCGCGGATCCGATGATATGGACGCCTTCG |
| 28a-PP_3227 a | TGGTGGTGCTCGAGGCGTTGCCTAAGACCGTATT |
| 28a-PP_3501 s | TGGGTCGCGGATCCATGACTTTCTCGCCCAATC |
| 28a-PP_3501 a | TGGTGGTGCTCGAGCAGTCAGAAGCGAGTTATGG |
| 28a-PP_4460 s | TGGGTCGCGGATCCATGGAGAACAGAATCACCCT |
| 28a-PP_4460 a | TGGTGGTGCTCGAGGCCTTCCACTCGTATCAGC |
| 28a-PP_5006 s | TGGGTCGCGGATCCGACTGGATGAAAACCCGC |
| 28a-PP_5006 a | TGGTGGTGCTCGAGGCTGCAACACTACATCTCCAG |
| T18-*cheA*_△domain_ s | CTAGTGGTGAATTCATGAGCTTCGGCGCCG |
| T18-*cheA*_△domain_ a | ATCTAGATCTCGAGCCACCACCAGGACCTTGA |
| T18-HPT^CheA^ s | CTAGTGGTGAATTCCGTTTGATGAGCTTCGGC |
| T18-HPT^CheA^ a | ATCTAGATCTCGAGTGTCACTTCGGCACGCT |
| T18-CheY Binding^CheA^ s | CTAGTGGTGAATTCAACAGCATGTTCGGCCAG |
| T18-CheY Binding^CheA^ a | ATCTAGATCTCGAGAGCGGGCTTGGCCACC |
| T18-Dimer^CheA^ s | CTAGTGGTGAATTCGAGAAGCACGCCGCCA |
| T18-Dimer^CheA^ a | ATCTAGATCTCGAGGCGGCCGAAGACCTTCT |
| T18-HATPase_c^CheA^ s | CTAGTGGTGAATTCACCGACCTTGACAAGAACCT |
| T18-HATPase_c^CheA^ a | ATCTAGATCTCGAGGTTGCCCAGCATCACCAT |
| T18-CheW Binding^CheA^ s | CTAGTGGTGAATTCGTCATCAAGGTGCCGCTG |
| T18-CheW Binding^CheA^ a | ATCTAGATCTCGAGCCGAAATCAAATACGCCG |
| 401-*cheA*_UP_ s | GGCCCCCCCTCGAGGAACTGAACCAGACCCGC |
| 401-*cheA*_UP_ a | GGCTGCAGGAATTCCCGAAGCTCATCAAACGT |
| 401-*cheA*_DW_ s | agcttcggGAATTCCGGCGTATTTGATTTCGG |
| 401-*cheA*_DW_ a | GAACTAGTGGATCCTGTGCTGGATCAACACGA |
| 401-*csoR*_UP_ s | GGCCCCCCCTCGAGCATTTGGCGGTCGGATTC |
| 401-*csoR*_UP_ a | GGCTGCAGGAATTCCGTGATCGCTCATGCCTT |
| 401-*csoR*_DW_ s | CGATCACGGAATTCTCACTAAGTACCTCTAGGCCTT |
| 401-*csoR*_DW_ a | GAACTAGTGGATCCGGGTTCGGGAATCTCGTC |
| MCS5-*csoR* s | GGCCCCCCCTCGAGACATGGTCGGCGGTCAG |
| MCS5-*csoR* a | GGCTGCAGGAATTCCCCAGCAGGATGGCACT |
| 402-*cheA* s | AGGAAACAGAATTCATGAGCTTCGGCGCCG |
| 402-*cheA* a | ACTCTAGAGGATCCCCACCACCAGGACCTTGA |
| 402-*csoR* s | AGGAAACAGAATTCATGAGCGATCACGAACAC |
| 402-*csoR* a | ACTCTAGAGGATCCCCAGCAGGATGGCACT |
| 402-*cheA*-*csoR* s | TCGAAAAAACTAGTATGAGCGATCACGAACAC |
| 402-*cheA*-*csoR* a | CACAGCGCGGCCGCCCAGCAGGATGGCACT |
| 402-*PP_5006* s | AGGAAACAGAATTCATGAAAACCCGCGATCG |
| 402-*PP_5006* a | ACTCTAGAGGATCCCGTCGGCTGCAACACTAC |
| 28a-GFPCheA s1 | GCGGCAGCCATATGATGGTGAGCAAGGGCGAG |
| 28a-GFPCheA a1 | GGCGAATTCGGATCCCTTGTACAGCTCGTCCATGC |
| 28a-GFPCheA s2 | GGATCCGAATTCGCCATGAGCTTCGGCGCCG |
| 28a-GFPCheA a2 | TGGTGGTGCTCGAGCCACCACCAGGACCTTGA |
| 28a-GFPCsoR s1 | GCGGCAGCCATATGATGGTGAGCAAGGGCGAG |
| 28a-GFPCsoR a1 | GGCGAATTCGGATCCCTTGTACAGCTCGTCCATGC |
| 28a-GFPCsoR s2 | GGATCCGAATTCGCCATGAGCGATCACGAACACG |
| 28a-GFPCsoR a2 | TGGTGGTGCTCGAGCCCAGCAGGATGGCACT |
| EMSA *copA-I*_pro_ s | TGTCAAGCTACCTGCTGAGCAGGATCGCCACAAGCA |
| EMSA *copA-I*_pro_ a | GCCGTAGGCAAACGCGTAG |
| Qpcr *copA-I* s | GCGTACAACTTCCACAAG |
| Qpcr *copA-I* a | TTGAGCAGGTAGGTGTAG |
| Qpcr *copA-II* s | CCAGCCTCAGAGACTAAC |
| Qpcr *copA-II* a | CAGATCCGCATAGGTCAG |
| Qpcr *copB-II* s | GTTGCAGCTTCTGTATGG |
| Qpcr *copB-II* a | GCCATTCTCTCCGATGAA |
| Qpcr *rpoD* s | CCTGATCCAGGAAGGCAACAT |
| Qpcr *rpoD* a | CAGGTGGCATAGGTCGAGAACT |
| *copA-I*_pro_-lacZ s | CTGATGCCGGTACCGCAGGATCGCCACAAGCA |
| *copA-I*_pro_-lacZ a | TTAGTCATCTGCAGGCCGTAGGCAAACGCGTAG |
| *copA-II*_pro_-lacZ s | CTGATGCCGGTACCCGATCAAAGCACTCGTAATG |
| *copA-II*_pro_-lacZ a | TTAGTCATCTGCAGTCTCGTGGTTTTGCTTTGC |
| *PP_0588*_pro_-lacZ s | CTGATGCCGGTACCGGCGATGCGGTATTTGTTG |
| *PP_0588*_pro_-lacZ a | TTAGTCATCTGCAGCATGCCTTGTACATTGAACACT |
| *csoR*_pro_-lacZ s | CTGATGCCGGTACCATGTGCGCCATTGTTGC |
| *csoR*_pro_-lacZ a | TTAGTCATCTGCAGGTGTTCGTGATCGCTCAT |
| 28a-CsoR_C40A_ s1 | TGGGTCGCGGATCCATGAGCGATCACGAACAC |
| 28a-CsoR_C40A_ a1 | GACGGCGGCCCGGCTGTCTTCGATCATGCTGAC |
| 28a-CsoR_C40A_ s2 | AGCCGGGCCGCCGTCGACATCGCTCAGCAACTG |
| 28a-CsoR_C40A_ a2 | TGGTGGTGCTCGAGCAGAACAGCCTGCTTCAC |
| 28a-CsoR_H65A_ s1 | TGGGTCGCGGATCCATGAGCGATCACGAACAC |
| 28a-CsoR_H65A_ a1 | GTGATCGATGGCGTCTTGGATAAGCACACGCTT |
| 28a-CsoR_H65A_ s2 | GACGCCATCGATCACTGCCTGGAACACACCGTC |
| 28a-CsoR_H65A_ a2 | TGGTGGTGCTCGAGCAGAACAGCCTGCTTCAC |
| 28a-CsoR_C69A_ s1 | TGGGTCGCGGATCCATGAGCGATCACGAACAC |
| 28a-CsoR_C69A_ a1 | CAGGGCGTGATCGATATGGTCTTGGATAAGCAC |
| 28a-CsoR_C69A_ s2 | ATCGATCACGCCCTGGAACACACCGTCGAAGC |
| 28a-CsoR_C69A_ a2 | TGGTGGTGCTCGAGCAGAACAGCCTGCTTCAC |
| 28a-GFPCsoR_C40A_ s1 | GCGGCAGCCATATGATGGTGAGCAAGGGCGAG |
| 28a-GFPCsoR_C40A_ a1 | GTCGACGGCGGCCCGGCTGTCTTCGATCATGCT |
| 28a-GFPCsoR_C40A_ s2 | CGGGCCGCCGTCGACATCGCTCAGCAACTGCAT |
| 28a-GFPCsoR_C40A_ a2 | TGGTGGTGCTCGAGCCCAGCAGGATGGCACT |
| 28a-GFPCsoR_H65A_ s1 | GCGGCAGCCATATGATGGTGAGCAAGGGCGAG |
| 28a-GFPCsoR_H65A_ a1 | ATCGATGGCGTCTTGGATAAGCACACGCTTGGC |
| 28a-GFPCsoR_H65A_ s2 | CAAGACGCCATCGATCACTGCCTGGAACACACC |
| 28a-GFPCsoR_H65A_ a2 | TGGTGGTGCTCGAGCCCAGCAGGATGGCACT |
| 28a-GFPCsoR_C69A_ s1 | GCGGCAGCCATATGATGGTGAGCAAGGGCGAG |
| 28a-GFPCsoR_C69A_ a1 | CAGGGCGTGATCGATATGGTCTTGGATAAGCAC |
| 28a-GFPCsoR_C69A_ s2 | ATCGATCACGCCCTGGAACACACCGTCGAAGCA |
| 28a-GFPCsoR_C69A_ a2 | TGGTGGTGCTCGAGCCCAGCAGGATGGCACT |
| T18-*csoR*_C40A/H65A/C69A_ s | CTAGTGGTGAATTCATGAGCGATCACGAACAC |
| T18-*csoR*_C40A/H65A/C69A_ a | ATCTAGATCTCGAGCAGAACAGCCTGCTTCAC |
| PHS-CheA(strep-tag II) s1 | GGATCCGAATTCGAGCTC |
| PHS-CheA(strep-tag II) a1 | GCTGCTGCCCATGGTATA |
| PHS-CheA(strep-tag II) s2 | CCATGGGCAGCAGCAGCCATATGAGCTTCGGC |
| PHS-CheA(strep-tag II) a2 | TCGAATTCGGATCCGGCGTAACGCTTGAGCAT |
